# MSCT study for adult esophageal diverticulum with secondary broncho-esophageal fistula

**DOI:** 10.1186/s13019-024-02510-7

**Published:** 2024-02-26

**Authors:** Xin Dong, Ruonan Pan, Lijun Duan, Xiaoqian Lu, Dianbo Cao

**Affiliations:** Department of Radiology, the First Hospital of Jilin University, No. 71 of Xinmin Street, Changchun, Jilin 130021 China

**Keywords:** MSCT, Broncho-esophageal fistula (BEF), Esophageal diverticulum, Endoscopy

## Abstract

**Background:**

Broncho-esophageal fistula (BEF) secondary to esophageal diverticulum is a rare clinical condition, which is often misdiagnosed for a long time. The aim of our study is to summarize and clarify the advantages of MSCT in diagnosing BEF secondary to esophageal diverticulum.

**Methods:**

We retrospectively analyzed patients clinically diagnosed with BEF from January 2005 to January 2022 at Jilin University First Hospital. Only those patients with BEF secondary to esophageal diverticulum and complete clinical data met our enrolled standard. All patients’ clinicopathologic characteristics and MSCT features were systemically evaluated.

**Results:**

17 patients were eligible for our cohort study, including male 10 and female 7. The patient’s mean age was 42.3 ± 12.5. The chronic cough occurred in all seventeen patients and bucking following oral fluid intake was documented in nine patients. MSCT distinctly suggested the fistulous tract between the bronchi and the esophagus in all patients. The mean diameter of the orifices in the wall of the esophagus was 4.40 ± 1.81 mm. The orifice in the midthoracic esophagus side was 15 cases and 2 cases at the lower thoracic esophagus. The involved bronchus included 13 cases at the right lower lobe bronchus, 1 at the right middle lobe bronchus and 3 at the left lower lobe bronchus. The contrast agent was observed in the pulmonary parenchyma in 10 of 13 patients who underwent esophagogram. No definite fistula was observed in 3 of 11 who underwent gastroscopy, while the intra-operative findings supported the existence of fistula.

**Conclusions:**

BEF secondary to esophageal diverticulum tends to occur between the midthoracic esophagus and the right lower lobe bronchus. Compared with esophagography and gastroscopy, MSCT shows more comprehensive information about the fistulous shape, size, course and lung involvement, which are helpful for establishing diagnosis and guiding subsequent treatment.

## Introduction

Broncho-esophageal fistula (BEF) is the pathological connection between the esophagus and the bronchus, which can be acquired or congenital. The BEF diagnosed in adulthood is usually acquired. Malignancies, most commonly esophageal cancer followed by lung cancer, account for more than half of cases in adults [1, 2]. Benign BEF caused by tuberculosis, injury, surgical operation, and tracheal intubation balloon compression are rare in adults [3]. Esophageal diverticulum is a blind-ended tube covered with epithelium that communicates with the esophageal lumen, and can be classified into congenital and acquired types based on various factors. Based on its pathogenesis, esophageal diverticula can be divided into traction and pulsion types. Traction diverticula are formed due to adhesion and traction of the esophageal wall caused by adjacent mediastinal lymphadenitis or granulomatous inflammation. They typically involve all layers of the esophageal wall, and are referred to as true diverticula. Sometimes, due to inflammatory necrosis, a fistula can occur between the esophageal diverticulum and the respiratory airways, or even a vascular structure [4]. In fact, BEF secondary to esophageal diverticulum is a rare clinical entity which can manifest as non-specific presentations such as mimicking pneumonia, bronchiectasis, prolonged coughing, and lung abscess. The presence of BEF has a severe impact on the health and survival of individuals due to prolonged dysphagia, bucking following oral fluid intake, recurrent aspiration pneumonia and weight loss [5]. Symptoms and signs of BEF vary according to different etiologies and the size, location, and shape of fistulous tract. Therefore, this disease entity is often misdiagnosed or missed for a long time.

Diagnostic methods for BEF include multi-slice spiral computed tomography (MSCT), upper gastrointestinal endoscopy, bronchoscopy, and contrast esophagogram. Among these imaging modalities, esophagogram with non-barium agent remains a preliminary radiographic investigation for the diagnosis of BEF. The shape, size and location of diverticula and fistulas can be easily visualized on esophagography. However, the image may sometimes be normal for orifices which are cabined owing to inflammation and edema. Although bronchoscopy can indicate the status of bronchus and obtain biopsy, its application is limited due to great pain to the patient. Upper gastrointestinal endoscopy is helpful in assessing the BEF, and large orifices in the wall of esophagus can be clearly seen. Unfortunately, the diagnosis of small fistulas hidden between the esophageal mucosal folds keeps not accurate [5]. MSCT is able to diagnose the BEF by directly or indirectly showing the course and shape of the fistulous tract between the esophagus and bronchus. Furthermore, MSCT scan is also capable of evaluating the lung and mediastinal involvements among those patients, which are unavailable in esophagogram and endoscopy. Meanwhile, it is the principal alternative in patients who are difficult to swallow, cooperate, tolerate fluoroscopy or endoscopy, like those patients with an altered mentation or on ventilator support. BEF that was previously not diagnosed or suspected can be further identified on MSCT scan for patients with recurrent respiratory symptoms.

Regarding this disease entity, most studies are only confined to limited case report in English literature. Therefore, our aim is to explore the advantages of MSCT in diagnosing BEF secondary to esophageal diverticulum, and provide a reference regarding therapeutic strategies for those patients with BEF.

## Materials and methods

**Fig. 1:**
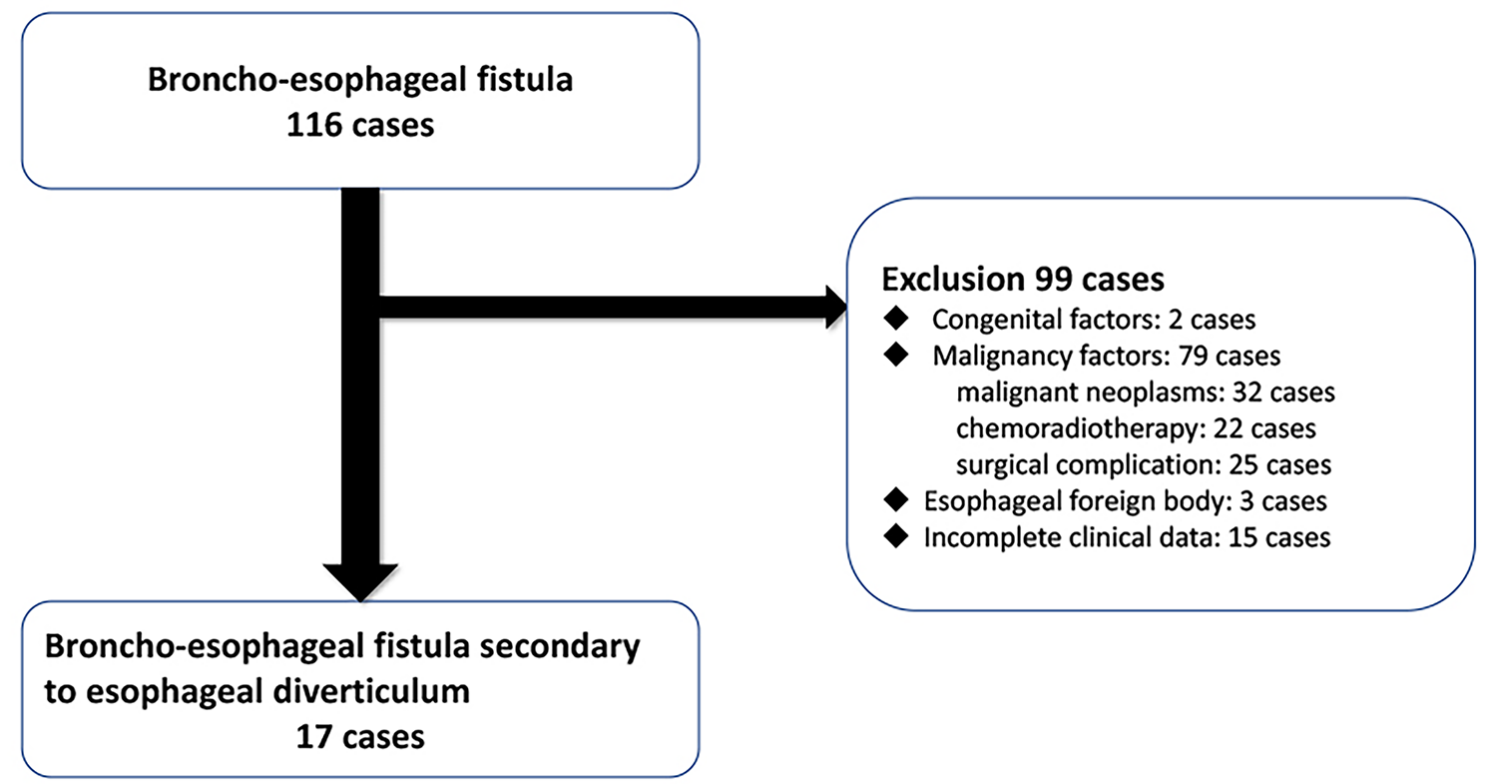
The patient selection process

Patients who were clinically diagnosed as broncho-esophageal fistula at The First Hospital of Jilin University were included into our retrospective study from January 2005 to January 2022. Patients with BEF caused by congenital factors, malignancies were excluded, such as congenital esophageal atresia and tracheoesophageal fistula, esophageal, bronchial or mediastinal neoplasms and their post-surgical pathology. In addition, patients with a history of esophageal trauma, esophageal rupture, or tracheoesophageal fistula were excluded. Patients under the age of 18 were also excluded. Those cases with unknown causes and incomplete clinical data were not enrolled into our cohort. BEF secondary to esophageal diverticulum met our criteria. Finally, 17 patients with BEF were eligible for our study (Fig. 1). All patients had complete clinical data and underwent MSCT scan.

16-slice, 64-slice, and 256-slice spiral CT were used for scanning patients. Before scanning, the patients received breath-hold training so as to guarantee consistency in the images. Contrast-enhanced scanning was performed with a high-pressure injector of bolus, with contrast flow velocity of 3.5 ml/s and a dose of 1.0 ml/kg calculated by patient weight. MSCT scan parameters were as follows: tube voltage, 120 kV; automatic tube current modulation (with reference mAs between 80 and 120mAs or similar noise levels). These thin-section images were reconstructed by using high-resolution algorithms (bone algorithm, high-resolution CT). All scans were photographed at windows and levels appropriate for the lung parenchyma (level, -550, -700 Hu; window, 1000–1500 Hu) and mediastinum (level, 30-60Hu; window, 300-500Hu). The slice reconstruction thickness was 1.0 or 1.5 mm. All rating was completed on a digital Picture Archiving System (PACS) workstation (Neusoft, China). 17 cases were read independently by two senior chest radiologists who were unaware of the diagnostic results. Interpretation discrepancy, if any, was resolved by consensus.

Our institutional review board waived the requirement to obtain written informed consent for this retrospective study, which evaluated de-identified data and involved no potential risk to patients. To avert any potential breach of confidentiality, no link between the patients and the researchers was made available.

## Results

A total of 17 patients were enrolled into our cohort study over the past 17 years. The average age was 42.3 ± 12.5 years (28–76 years), including male 10 and female 7. Clinical data and presentations of patients were systematically described (Table 1). The chronic cough and expectoration occurred in all seventeen populations. Eleven patients (11/17) presented with fever, six patients (6/17) had hemoptysis. Remarkably, bucking following oral fluid intake was prominent in nine patients, which led to malnutrition and weight loss. Two patients complained of chest pain and another one had the presentation of long-termed dyspnea due to fear of having a meal. The duration of symptoms in these patients ranged from one month to two decades.

**Table 1:** Clinical characteristics of included patients

Characteristics	No. of patients (*n* = 17)	Percentage (%)
Age, mean (range)	42.3 ± 12.5 (28–76)	
Sex		
Male	10	58.8
Female	7	41.2
Clinical manifestations		
Chronic cough and expectoration	17	100
Fever	11	64.7
Hemoptysis	6	35.3
Cough after choking on water	9	52.9
Chest pain	2	11.8
Dyspnea	1	5.9

All patients underwent MSCT scans with thin constructed images. Two patients underwent contrast-enhanced MSCT and four patients immediately underwent MSCT after oral contrast esophagography. The abnormal tract between bronchus and esophagus could be distinctly identified on MSCT images of each patient. Only one fistula was observed per patient and the mean diameter of the orifice in the esophageal wall was measured as 4.40 ± 1.81 mm. Esophageal dilatation occurred in nine patients (9/17; 52.9%) due to the presence of large amounts of gas retention in the esophagus. The orifice of fistulas was mainly situated in the mid-thoracic esophagus (15/17; 88.2%), followed by the lower thoracic esophagus (2/17; 11.8%). The involved bronchus included the right lower lobe bronchus (13/17; 76.5%), the left lower lobe bronchus (3/17; 17.6%) and the right middle lobe bronchus (1/17; 5.9%). It could be found aggregation of air or fluid in the airways and the thickening walls of the esophagus (6/17; 35.3%) and the trachea (12/17; 70.6%) on MSCT images, which were probably caused by persistent inflammatory process. All patients had lung parenchymal and interstitial changes associated with aspiration pneumonia or secondary infection. The abnormalities of lung included bronchiectasis (15/17; 88.2%), consolidation (11/17; 64.7%), ground glass opacity (9/17; 52.9%) and scattered pulmonary emphysema (2/17; 11.8%). In addition, pleural thickening (6/17, 35.3%) on the affected side could be noticed on MSCT images of patients, two of whom also had pleural effusion simultaneously. It was a typical fact that pneumonia occurred close to the spine in almost all patients (15/17; 88.2%). The patients’ detailed data regarding MSCT findings were summarized in Table 2.

**Table 2:** CT imaging features of broncho-esophageal fistula secondary to esophageal diverticulum

Imaging feature	No. of patients (*n* = 17)	Percentage (%)
Direct connection identified	17	100
Diameter of fistula, mean(mm)	4.40 ± 1.81	
Esophageal dilation with air	9	52.9
Esophageal wall thickening	6	35.3
Location of fistula on the esophageal side		
Mid-thoracic esophagus	15	88.2
Lower thoracic esophagus	2	11.8
Involved bronchus		
Right middle lobe bronchus	1	5.9
Right lower lobe bronchus	13	76.5
Left lower lobe bronchus	3	17.6
Airway wall thickening	12	70.6
Pulmonary abnormality		
Bronchiectasis	15	88.2
Consolidation	11	64.7
Ground glass opacity	9	52.9
Scatter pulmonary emphysema	2	11.8
Pleural thickening	6	35.3
Pleural effusion	2	11.8
Location of pneumonia		
Para-spine	15	88.2
Other locations	2	11.8

**Fig. 2:**
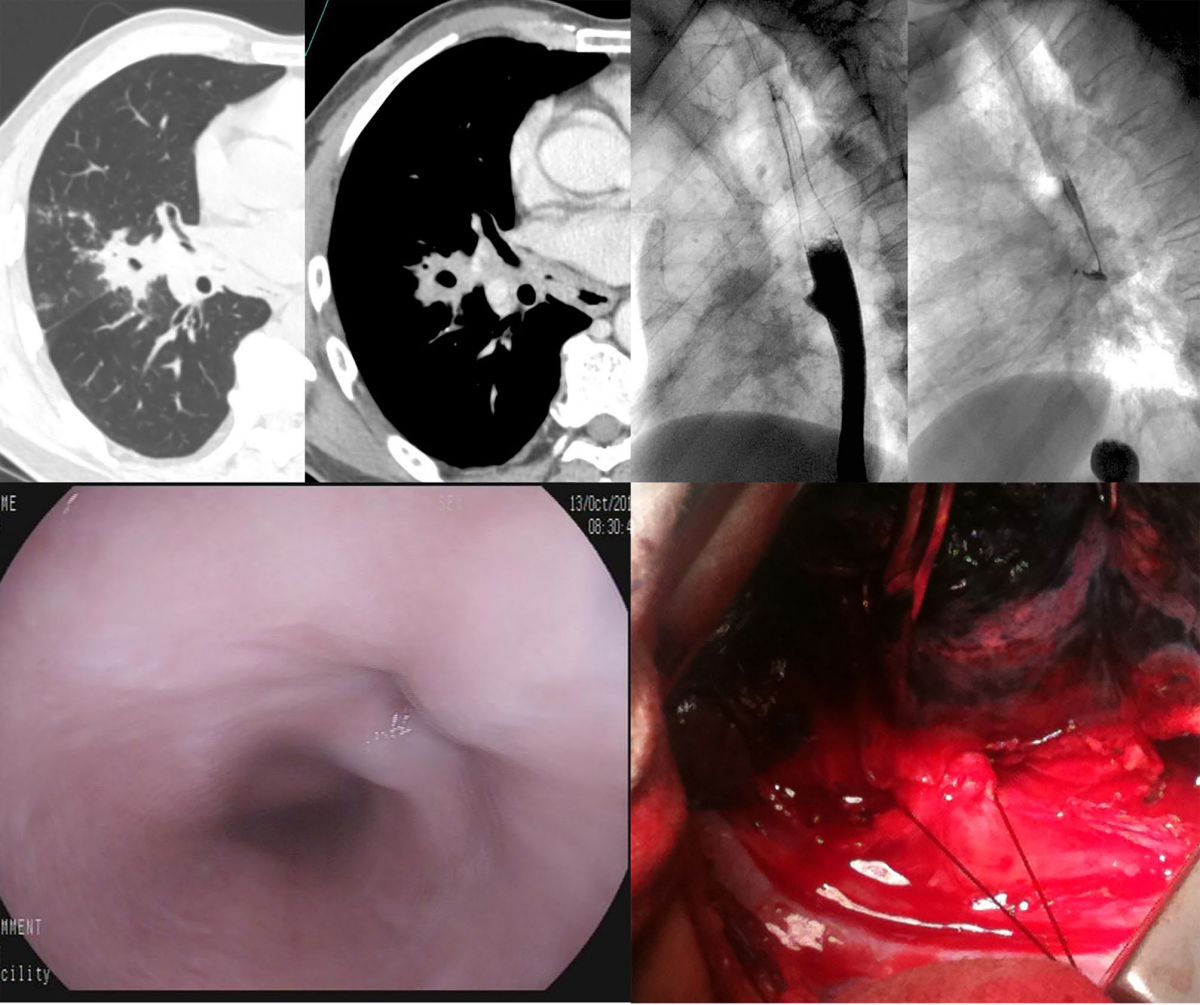
Funnel-shaped fistula; MSCT, contrast esophagogram and gastroscopy before surgery. Fistula observed during surgery

**Fig. 3:**
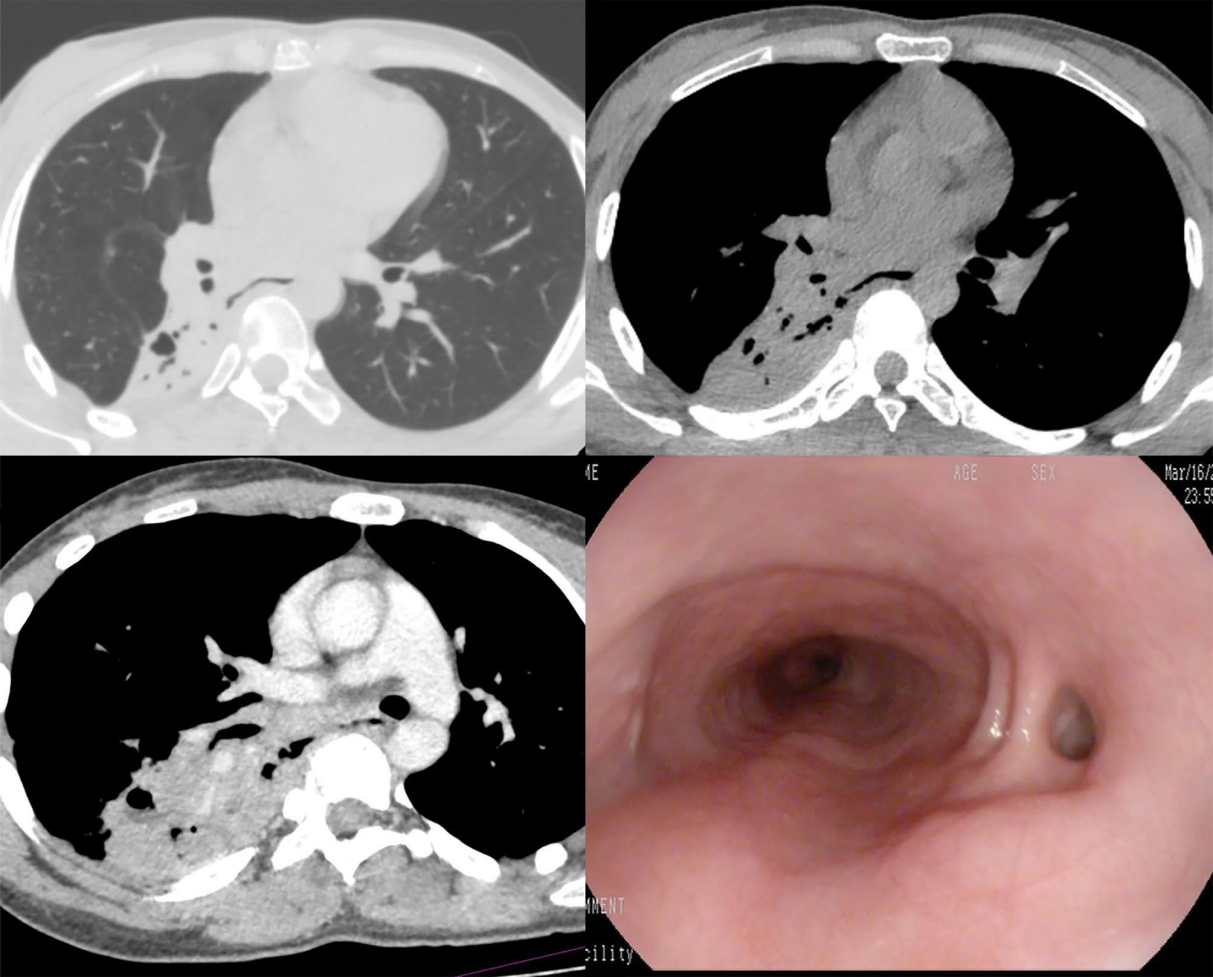
Columnar fistula; MSCT and contrast-enhanced CT images. Fistula observed by gastroscopy

Esophagography was performed in thirteen patients, and eleven patients underwent upper gastrointestinal endoscopy. The contrast agent was observed in the pulmonary parenchyma in 10 of 13 patients, with a funnel-shaped fistula in 7 patients(Fig 2), columnar in 2 patients(Fig 3) and irregular in 1 patient. No definite fistula was present in 3 of 11 patients who underwent endoscopy, while intra-operative findings demonstrated the existence of fistula. A comparison of the diagnostic performance of MSCT with esophagography and endoscopy was summarized (Table 3).

**Table 3:** The comparison of the diagnostic performance of different investigations for BEF

Variables	No. of patients	Percentage (%)
Esophagography		
Total	13	
Positive	10	76.9
Funnel-shaped fistula	7	70
Columnar fistula	2	20
Irregular fistula Negative	1 3	10 23.1
Upper gastrointestinal endoscopy		
Total	11	
Positive	8	72.7
Negative	3	27.3
Multi-slice spiral computed tomography		
Total	17	
Positive	17	100
Negative	0	0

## Discussion

BEF, which manifests as a direct channel between the esophagus and bronchus, is commonly caused by congenital malformations or pathological factors such as malignancy, tuberculosis, candidiasis, trauma and so on [6]. BEF associated with esophageal diverticulum is a rare clinical entity with only a few case reports in previous studies. Esophageal diverticulum is a blind-ended tube covered with epithelium that communicates with the esophageal lumen and can be congenital or acquired due to various factors [3]. Based on their pathogenesis, esophageal diverticula can be categorized into traction and pulsion types. Pulsion-type esophageal diverticula result from increased intraluminal pressure within the esophagus, causing partial protrusion of the esophageal mucosa and submucosa through a weakened point in the muscular layer. These diverticula do not involve the entire esophageal wall and are referred to as false diverticula. Traction-type diverticula, on the other hand, develop as a result of adjacent mediastinal lymphadenitis or granulomatous inflammation causing adhesion and traction on the esophageal wall [7]. They typically involve all layers of the esophageal wall and are termed true diverticula. The relationship between underling esophageal motility disorders and esophageal diverticulum formation has also been highlighted in previous studies [8, 9]. When an esophageal diverticulum, due to diverticulitis or chronic inflammation with adhesion and traction on the esophageal wall, leads to chronic perforation and connection with the adjacent respiratory tract, it can result in mediastinitis or BEF. In short, it is believed that esophagobronchial fistula secondary to esophageal diverticulum is a result of an inflammatory necrosis process that leads to the formation of a passage between the esophagus and the bronchus [4]. According to our study, most diverticula occur in the mid-thoracic esophagus and lead to serious consequences, and a small minority in the low thoracic esophagus. This is closely related to its anatomy, because the lungs are immediately beside the esophagus at this level [10]. In general, these diverticula have no specific clinical manifestations and no intervention is necessary. Nevertheless, severe complications such as hemorrhage, perforation or fistulous formation with adjacent bronchi may occur due to chronic inflammation and tissue necrosis [7, 11]. After the formation of fistula, some components will enter the lung parenchyma through the fistulous tract when the patient ingests non-solid food. Prolonged aspiration results in recurrent pneumonia and related clinical presentations such as chronic cough, fever and hemoptysis, which will seriously affect the patients’ life quality.

The manifestations of BEF are complicated and variable, which can remain asymptomatic for a long interval, as described in our patients. Chronic coughing related to swallowing is known as the common symptoms of BEF [11], which is consistent with our outcome. In our cohort, 52.9% (9/17) patients presented with choking following oral fluid intake, all of whom developed repeated pulmonary infections. When the fistula remains cabined or the small orifice due to inflammation and impaction of esophageal contents, food and fluid from gastro-esophageal reflux are not easy to flow into bronchus through the passage, which explains why some patients have no or mild pneumonia. Gastro-esophageal reflux is the most damaging to the respiratory system, resulting in bronchial obstruction, atelectasis, Mendelson syndrome and/or dyspnea, which is also a significant cause of mortality in patients [12]. Fever was observed in 64.7% (11/17) cases, probably because of pulmonary infection. Repeated fever, choking cough and poor appetite due to aspiration usually lead to malnutrition and weight loss. In our study, 35.3% (6/17) patients demonstrated the presentation of hemoptysis due to bronchiectasis, which had the potential risk to induce fatal massive hemoptysis. Svane et al. reported that an 80-year-old woman with BEF died of massive hemoptysis in previous studies [13]. In short, the clinical manifestations of BEF remain complex and changeable, so the timely and accurate diagnosis is of great significance for the prognosis of those affected patients. The diagnosis of BEF should be alerted when the patient develops coughing while drinking and eating, recurrent pneumonia neighboring the mid-lower thoracic spine.

Computed tomography, upper gastrointestinal contrast, gastroscopy and bronchoscopy are helpful in the diagnosis of BEF. The chest radiography may appear normal in the initial stage, but will show pulmonary complications including pneumonia, pulmonary infiltration and ARDS if reflux contents enter the lung through the fistula [5]. However, no patient presenting with ARDS was present in our cohort. Other non-specific manifestations such as esophageal inflation and scattered pulmonary emphysema may be helpful in the diagnosis, but chest radiography is limited informative. Esophagogram with oral contrast agent is helpful to accurately depict the location of fistula and the contrast-contained tract between the esophagus and the bronchus. Unexpectedly, contrast agent into the bronchi and/or lung was present in ten of thirteen patients with esophagogram. It has been familiar that BEF can be definitely diagnosed by spread of oral contrast material to the adjacent airway outside the esophagus, but the absence of this phenomenon does not completely rule out the existence of fistulas [14]. In fact, there remains a number of reasons why the contrast medium is not visible. The characteristics of the fistula itself, such as the small size of the orifice, the blockage of the inner mouth or the acute angle between the fistula and the esophageal wall, may be the key reason affecting contrast medium into the lung. For the rest, the surrounding tissue of the fistula, such as compression of the mediastinal tissue, the contraction and obstruction of the esophageal mucosal folds may lead to temporary closure of the tract and accounts for false negative results. Additionally, the check posture of oral contrast medium may also affect the results, which the upright position is not effective as the supine or lateral positions [14]. In addition to focusing on the condition of contrast medium into the lung, we also analyzed the relationship between fistulas and esophageal diverticula. Esophagography showed that the fistulas were funnel-shaped in seven cases, columnar in two cases and irregular in one case. The combination of a small orifice and a large diverticulum forms a funnel-shaped fistula, however, dilated ducts that communicate with diverticula or the large orifice may be the main cause of cylindrical fistulas.

Endoscopy remains the effective diagnostic option to detect and evaluate patients with BEF. Bronchoscopy and gastroscopy offer a direct visualization of the BEF from different views, locating the position of the BEF, observing the mucosal changes around the fistula and taking sample for pathological analysis. Notably, the large fistula can be easily visualized by gastroscopy, but the accuracy is reduced if the fistula is hidden between the esophageal mucosal folds [5, 6, 11]. In our study, no definite fistulas were found in 3 of 11 cases that underwent upper gastrointestinal endoscopy, which might be caused by the above factors.

MSCT plays an important role in the diagnosis of BEF, lung assessment and therapeutic guidance. MSCT is capable of demonstrating the direct or indirect connection between the esophagus and the airway. If the patient implements a MSCT scan following oral contrast agent, intrapulmonary attenuation of contrast agent can be easily seen on the mediastinal window. MSCT is not only able to directly display the fistula based on thinned continuous images, but also demonstrates the fistula in multiple planar reconstruction and conducts a comprehensive evaluation for the fistulous tract and adjacent tissue. Several indirect signs, such as pulmonary changes, tracheal and esophageal abnormalities, are also helpful in the diagnosis of BEF when the fistulous tract is intermittent shadow on consecutive MSCT images. In our cohort, pulmonary complications include bronchiectasis, consolidation, ground glass opacity, and scattered pulmonary emphysema, which were closely associated with aspiration pneumonia. Most patients have thickening of the esophageal and tracheal walls and esophageal dilatation, which can also be manifested as indirect signs for the diagnosis of BEF. Of note, the diagnosis of BEF should be highly suspected when pneumonia indistinct with the esophagus on the para-vertebral area is observed on MSCT investigation. In addition, MSCT scan remains better than esophagography and endoscopy in assessing intrapulmonary and mediastinal changes because of the complete visualization for esophageal surrounding structures. Six of seventeen patients with lung parenchymal abnormalities had pleural thickening, and two pleural effusions. Early and accurate diagnosis and therapeutic intervention based on the clinical presentations, radiographic findings and endoscopic evaluation will avoid catastrophic consequences in those patients with BEF. The location and course of the fistula delineated by MSCT are of importance to guide the surgical approach [15, 16]. In order to reach a good surgical outcome, patients with BEF must be properly prepared prior to surgery. Pulmonary infections caused by aspiration must be controlled via the administration of effective antibiotics, and supportive therapy should also be given to improve patients’ nutritional status. Simultaneously, preoperative pulmonary MSCT assessment can also reveal insight regarding infectious control.

The limitation of our study is that the registered individuals were all from the same unit, which may lead to less generalization of the results. Secondly, our study cohort was small due to the rare populations with BEF secondary to the esophageal diverticulum. In future studies, more clinical data should be collected from multiple centers to assess the sensitivity and specificity of MSCT examination.

## Conclusion

MSCT as an effective radiographic method plays an essential role in the diagnosis and evaluation of BEF secondary to the esophageal diverticulum. BEF tends to occur between the mid-thoracic esophagus and the right lower lobe bronchus. MSCT is able to reveal fistula that is difficult to be detected on esophagogram or endoscopy. Compared with esophagography and endoscopy, MSCT shows more comprehensive information about the fistulous shape, size, course and the extent of lung involvement.

## Data Availability

Data sharing is not applicable to this article as no datasets were generated or analysed during the current study.
